# *Altrichthys alelia*, a new brooding damselfish (Teleostei, Perciformes, Pomacentridae) from Busuanga Island, Philippines

**DOI:** 10.3897/zookeys.675.12061

**Published:** 2017-05-18

**Authors:** Giacomo Bernardi, Gary C. Longo, T.E. Angela L. Quiros

**Affiliations:** 1 Department of Ecology and Evolutionary Biology, University of California Santa Cruz, California, USA; 2 Current address: Conservation Biology Division, Northwest Fisheries Science Center, National Marine Fisheries Service, National Oceanic and Atmospheric Administration, Seattle, WA, USA

**Keywords:** Apelagic fishes, *Acanthochromis*, CO1, Control region, RAD markers

## Abstract

A new species of damselfish, *Altrichthys alelia* **sp. n.** is described from specimens collected in shallow water (1–8m depth) off Busuanga Island, Palawan Province, Philippines. It differs from the other two species in the genus, *A. curatus* and *A. azurelineatus*, in various features including having golden upper body lacking dark edges of dorsal and caudal fins, higher modal number of tubed lateral line scales, as well as differences in two mitochondrial markers, one nuclear marker, and RAD markers.

## Introduction

**Figure 1.**
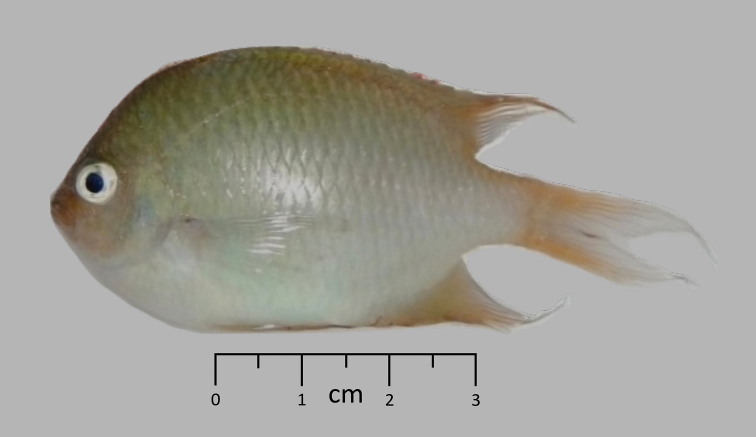
Holotype of *Altrichthys alelia*, shortly after capture. Photo by Gary C. Longo.

The damselfish genus *Altrichthys* Allen, 1999 includes two species, the azure damselfish, *Altrichthys azurelineatus* (Fowler & Bean, 1928), and the guardian damselfish, *Altrichthys curatus* Allen, 1999, that occur on shallow coral reefs in the Calamian Archipelago, northern Palawan Province, Philippines (Allen 1999, Bernardi 2011, Bernardi et al. 2017). While conducting exploratory dives in the less-studied area of northern Busuanga Island, in the region of San José, we observed and collected *Altrichthys* individuals that after closer examination and laboratory work showed unique morphological and genetic characters that distinguish them from previously described *Altrichthys* species. We can therefore confirm the presence of a third *Altrichthys* species that we hereby describe as the new species *Altrichthys alelia* (Figure 1).

## Methods and materials

Specimens were collected with hand nets while free or scuba diving in less than 8m depth. Counts and measurements follow Allen (1999). Measurements were made days to a few weeks after collection and were taken to the nearest 0.1 mm with digital calipers. Collection abbreviations are as follows: PNM = National Musem of the Philippines, CAS = California Academy of Sciences.

**Figure 2.**
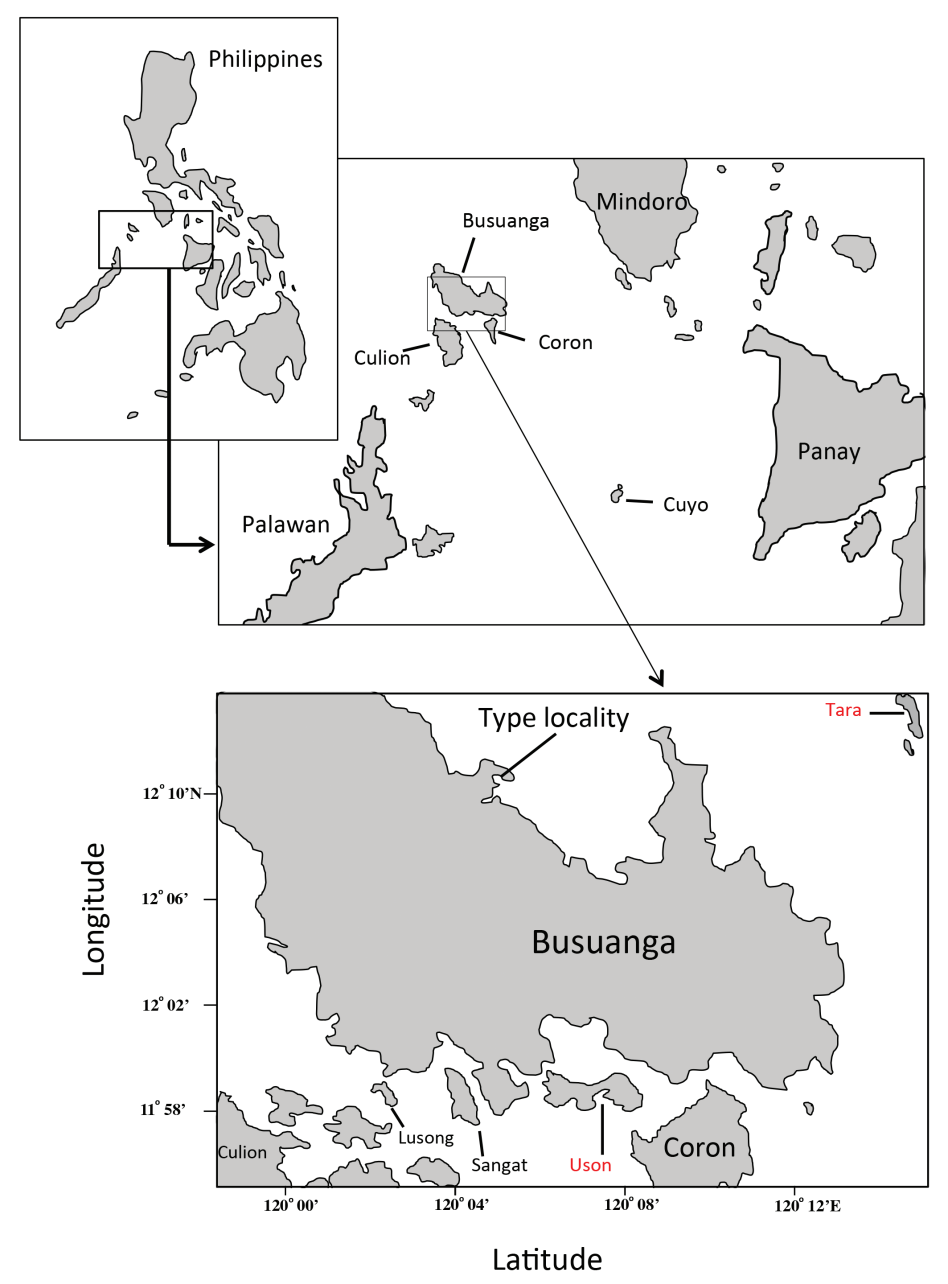
Type locality of *Altrichthys alelia*. Busuanga Island, Palawan Province, Philippines, near the village of Maricaban, next to the resort “El Rio y Mar”, 12.1911°N; 120.1022°E. Highlighted in red are Tara Island and Uson Island, where specimens of *Altrichthys* were first collected, as well as Uson Island and Sangat Island where sister species *A. azurelineatus* and *A. curatus* were collected.

Fin clip tissue samples were stored in 95% ethanol and DNA was extracted using DNeasy Blood & Tissue kits (Qiagen) according to the manufacturer’s protocol. DNA was extracted from two *Altrichthys alelia* individuals (juvenile individuals 6 and 8, in CAS 241439), and from five individuals of each sister species, *A. azurelineatus* and *A. curatus* collected at Uson Island and Sangat Island (Figure 2). Extractions were PCR amplified for two mitochondrial (control region and cytochrome oxidase 1) and one nuclear marker (RAG2) following published protocols (Bernardi 2011) (Genbank accession numbers KY963970- KY963994; KY969587, KY969588). Phylogenetic reconstructions were done based on the Neighbor-Joining method generated in R (R Core Team 2013) with the ape package (Paradis et al. 2004) using Kimura-2 parameter substitution models, and a Maximum Likelihood method implemented in GARLI (Zwickl 2006). Node support was obtained using 1000 bootstrap replicates and retaining values that support nodes in more than 50% of the bootstrap replicates.

In addition, we constructed RAD libraries using a variation of the original protocol with restriction enzyme SbfI (Miller et al. 2007, 2012, Baird et al. 2008, Longo and Bernardi 2015, Omar et al. 2016). Individually barcoded samples were sequenced on an Illumina HiSeq 2500 at the Vincent J. Coates Genomics Sequencing Laboratory at UC Berkeley. Analysis of the sequences followed previously published protocols (Longo and Bernardi 2015).

## Taxonomy

### Alelia’s damselfish

#### Altrichthys alelia

Taxon classificationAnimaliaPerciformesPomacentridae

Bernardi, Longo, & Quiros sp. n.

http://zoobank.org/0C9577DE-0F3F-4B27-80F7-9E1B4725212B

##### Type locality.

San José, Busuanga Island, Philippines, 12.1911°N ; 120.1022°E.

##### Holotype.

PNM 15195; 54.7 mm S.L., San José, Busuanga Island, Philippines, 12.1911°N ; 120.1022°E, 3m, hand net, G. Bernardi, G. Longo and A. Quiros (Figures 1, 2).

##### Paratypes.

CAS 241438, 3 adult specimens, SL 51.0-54.1 mm; CAS 241439, 8 juvenile individuals, SL 13.9 – 19.7mm. Both lots collected with Holotype, Busuanga Island, Philippines, 3m, hand net, G. Bernardi, G. Longo and A. Quiros.

##### Comparative material.

*Altrichthys azurelineatus*. Holotype: USNM 89957 (one specimen, Uson Island), Paratypes USNM 96398 (one specimen Tara island), USNM 96425 (one specimen, Tara Island). *Altrichthys azurelineatus* 5 specimens from Uson Island, *A. curatus* 5 specimens from Uson Island and Sangat Island.

##### Diagnosis and description.

**Figure 3.**
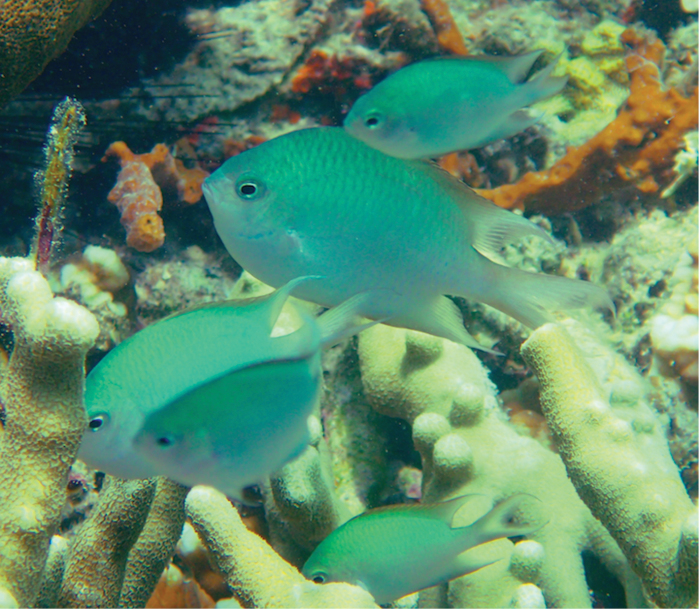
*Altrichthys alelia* in its natural environment, near a common nesting substrate, the coral *Porites cylindrica*.

**Figure 4.**
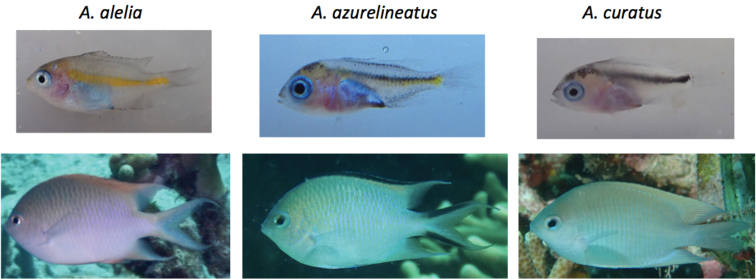
*Altrichthys* species. Juvenile and adult *Altrichthys* are shown from left to right: *A. alelia*, *A. azurelineatus*, and *A. curatus*. Note the prominent yellow line in the juvenile *A. alelia*, and the lack of black margins in the dorsal and caudal fins in the adult.

A species of *Altrichthys* distinguished by the following combination of characters: dorsal rays XIV, 13–14; anal rays II, 15, tubed lateral line scales 14–15 (Table 1); preorbital and sensory pores small and numerous, usually more than 30, adult coloration in life pale green on upper half grading to white on lower part; iris silvery; pale yellow to gold outer margin of dorsal and upper and lower edges of caudal fin. Fins mainly white to translucent. Juveniles up to 16mm in length are mostly white with a prominent yellow stripe along the lateral line (Figure 3). Adults are generally of the same size as other *Altrichthys* adults, approximately 70–80mm TL. *Altrichthys alelia* differs from *A. curatus* by having long filaments at the trailing edges of the dorsal and caudal fins, and from *A. azurelineatus* by lacking any black lining of the outer edges of dorsal and caudal fins. These black margins are represented by yellow/gold margins in *A. alelia* (Figure 4). Pored lateral line scales easily distinguish *A. curatus* (17–18) and *A. azurelineatus* (10-14). Counts for *A. alelia* are most similar to and overlap *A. azurelineatus* counts, but exhibit a higher mode (15).

**Table 1.** Morphometric and Meristic measurements of *Altrichthys*. Lines present, from top to bottom, numbers for Total Length (TL), Standard Length (SL), Depth, With, Eye Diameter (Eye), spine and ray counts for Dorsal and Anal fins, and Tubed Lateral Line Scales (Tubed LL scales). Number in parenthesis represent percentage of the Standard length). The first column is the species holotype.

Voucher number	PNM 15195/	CAS 241438/	CAS 241438/	CAS 241438/
	9	10	11	12
TL (mm)	73.6	66.8	66.2	70.2
SL (mm)	54.9	51.6	51.0	54.1
Depth (mm)	31.6 (57.6%)	30.2 (58.5%)	30.0 (58.8%)	31.2 (57.7%)
Width (mm)	9.6 (17.5%)	11.0 (21.3%)	11.6 (22.7%)	11.1 (20.5%)
Eye (mm)	5.2 (9.5%)	5.4 (10.5%)	5.6 (11.0%)	5.7 (10.5%)
Dorsal fin	XIII, 14	XIV, 13	XIV, 13	XIII, 14
Anal fin	II,15	II,15	II,15	II,15
Tubed LL scales	15	14	14	15

**Figure 5.**
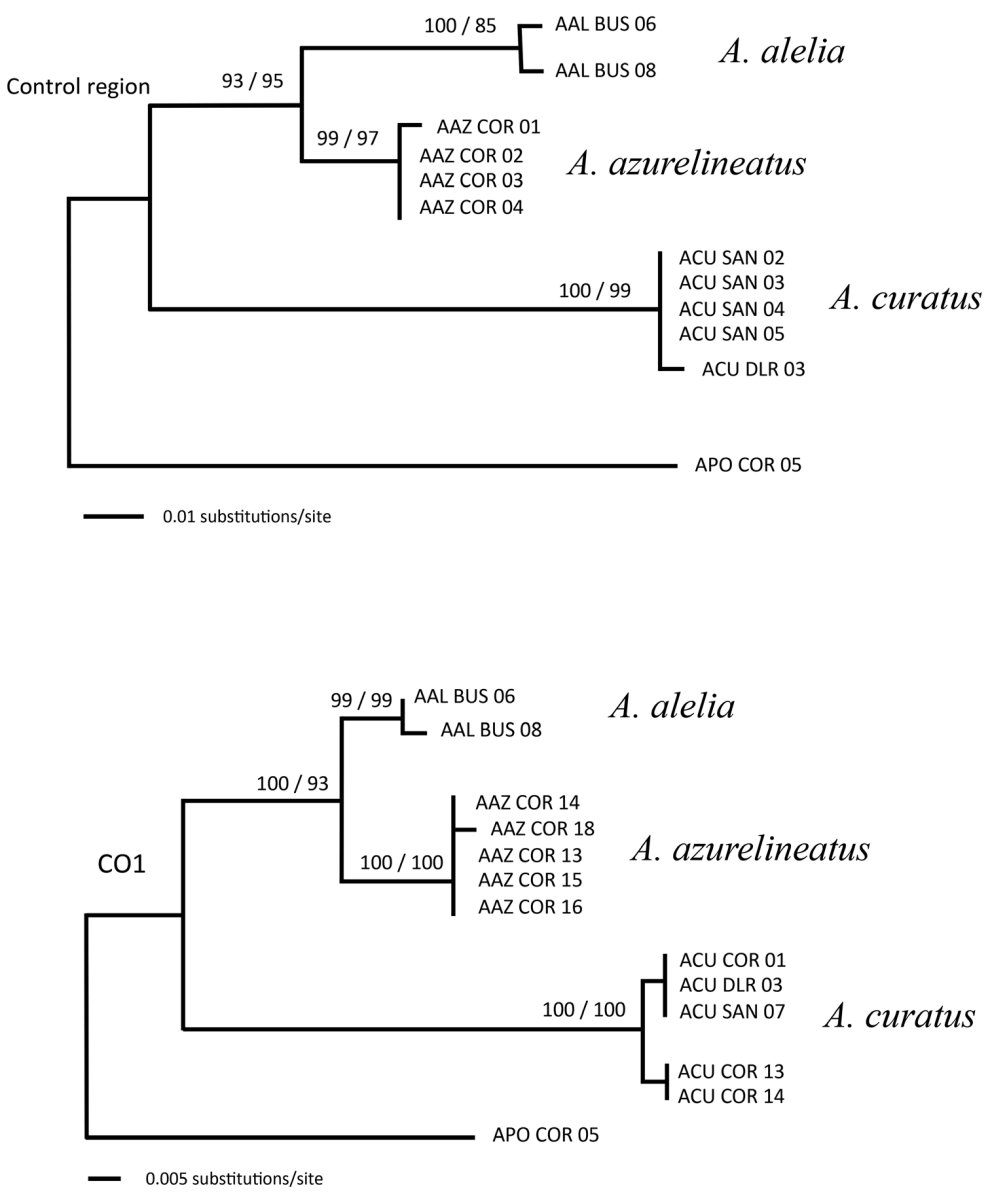
Phylogenetic reconstructions of *Altrichthys* species based on mitochondrial control region (313 aligned base pairs) and cytochrome oxydase 1 (CO1, 611 aligned base pairs). The closely related *Acanthochromis polyacanthus* was used as an outgroup. Sample labels refer to collection sites: BUS, El Rio y Mar Resort, Busuanga Island, COR, Balinsasayaw Resort, Uson Island; SAN, Sangat Island; DLR, Dive Link Resort, Uson Island. Numbers above nodes refer to percent of 1000 bootstrap replicates used in Neighbor-Joining (left) and Maximum Likelihood (right) reconstruction methods.

In addition, Sanger sequencing results show that *Altrichthys alelia* individuals form a group most closely related to, but distinct from, *A. azurelineatus* (Figure 5). *Altrichthys alelia* sequences differed from their closest relative *A. azurelineatus* by one fixed difference at the nuclear locus RAG2. For mitochondrial markers, *Altrichthys alelia* sequences differed from *A. azurelineatus* by 13 and 15 fixed mutations for cytochrome oxidase I and control region markers, respectively, thus corresponding to a Kimura-2 sequence divergence of 2.3% and 5.9% respectively. These divergences are consistent with values obtained in other sister species of fish (Ward et al. 2005). RAD DNA sequencing results were also consistent with *A. alelia* and *A. azurelineatus* being distinct species and more closely related to each other than either are to *A. curatus* (Table 2). Indeed, RAD sequencing generated 8383 variable SNPs for the 5 sequenced individuals (2 *A. alelia*, 1 *A. azurelineatus*, 2 *A. curatus*). Of those 8383 SNPs, 1584.5 (18.9%) showed differences between *A. alelia* and *A. azurelineatus*; while 7224.5 (86.2%) and 6495 (76.4%) showed differences between *A. curatus* and *A. alelia* and between *A. curatus* and *A. azurelineatus*, respectively (Table 2).

**Table 2.** Results of RAD sequencing in three species. Results are based on 8383 variable SNPs for 5 sequenced individuals (2 *A. alelia*, 1 *A. azurelineatus*, 2 *A. curatus*). Figures refer to the number of SNPs and their percentage (over 8383 total SNPs).

	*A. alelia*	*A. azurelineatus*	*A. curatus*
*A. alelia*	123 / 1.47%		
*A. azurelineatus*	1584.5 / 18.9%	– / –	
*A. curatus*	7224.5 / 86.2%	6495 / 76.4%	8 / 0.0%

##### Distribution.

Known from northern Busuanga Island at San José, Palawan Province, Philippines (Figure 2).

##### Habitat.

Collected off live and extensive thickets of corals mostly *Porites cylindrica*.

##### Etymology.

The name *Altrichthys alelia* derives from the combined first names of Alessio Bernardi and Amalia Bernardi, who greatly helped during field-work on *Altrichthys*.

##### Common name.

We suggest Alelia’s damselfish as a literal translation of the scientific name.

## Discussion

Species of the genus *Altrichthys* together with their close relative *Acanthochromis polyacanthus* are unusual as they brood their young (Allen 1999, Bernardi et al. 2017), whereas most coral reef fishes have a pelagic larval stage (Leis 1991). This behavior is clearly visible on the reef, by the presence of pairs of adult fish aggressively defending a cloud of fry that remain around the parents. An identical behavior was observed in *A. alelia*, where fry and adult pairs were associated, and similarly to the other *Altrichthys* species, *Porites cylindrica* is used as a nesting substrate (Bernardi et al. 2017). While *Altrichthys azurelineatus* and *A. curatus* co-occur at most locations in southern Busuanga, we did not observe any other *Altrichthys* species occurring in the vicinity of *A. alelia*.

**Figure 6.**
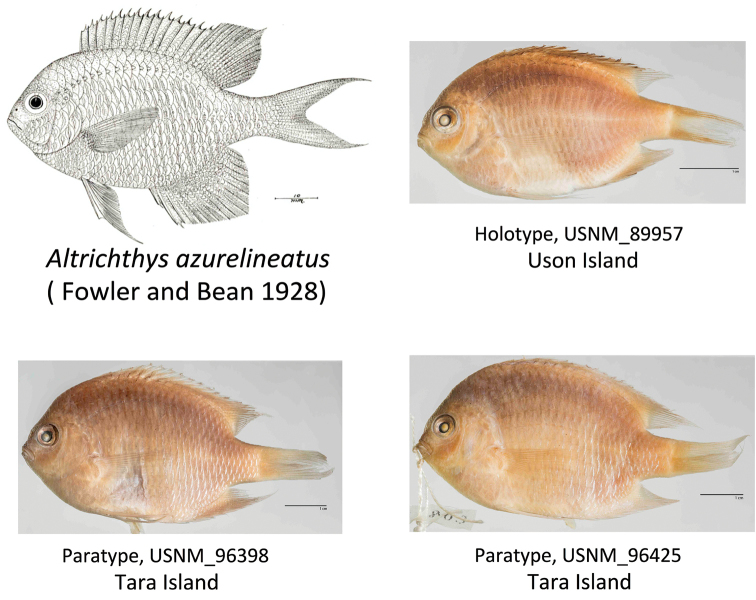
Drawing (upper left, from Fowler and Bean 1928) and photo (upper right) of the holotype of *Altrichthys azurelineatus* from Uson Island (USNM 89957), and paratypes (bottom photos) (USNM 96398, 96425) collected at Tara Island. Photos by Sandra J. Raredon, Division of Fishes, Smithsonian Institution.

*Altrichthys alelia* may have been observed previously by other scientists, but remained unnoticed. The original description of *Chromis azurelineatus* (Fowler & Bean, 1928), later re-described as *Altrichthys azurelineatus* (Allen, 1999), was based on fish collected during the Albatross expedition of 1908-1909. Three collections were made in December 1908 (Smith and Williams 1999). The collection that yielded the type specimen was made in Uson Island, using dynamite. The authors spent several days on Uson Island, and observed hundreds of *A. azurelineatus* there but never saw *A. alelia* in that locality. That specimen indeed looks like a genuine *A. azurelineatus* with its distinctive dark caudal fin margins (USNM, 89957, Figure 6, top right panel). The other collections of the Albatross were at Tara island, which faces the north-eastern portion Busuanga Island, a region close to the type locality of *A. alelia* (Figure 2). Fish collected at Tara island have “dorsal spines bright orange, color carried somewhat into membranes in another portion of the fin” (Fowler and Bean 1928). The geographic location and description are consistent with *A. alelia*, however the lack of diagnostic morphological and meristic characters precludes final determination if this were the case. Nevertheless, specimens from these collections lack the characteristic black margins on the caudal fin that is found in *A. azurelineatus* (USNM, 96398, and USNM, 96425, Figure 6, bottom left and bottm right panels). The drawing of *A. azurelineatus* in the original description by Fowler and Bean (1928), and later redrawn in color (Allen 1991), does not show the dark margins either (Figure 6, top left panel), as it may have been a compilation of all the various specimens collected (i.e. a combination of *A. azurelineatus* and *A. alelia* individuals). As such, that drawing is more similar to *A. alelia*. Finally, the picture taken on Busuanga island labeled as *A. curatus* in Figure 2 of Allen (1999), looks precisely like *A. alelia*. Due to the peculiarities of this fish, Allen (1999) suggested that “additional observations are needed”, which is what we are presenting here.

## Supplementary Material

XML Treatment for Altrichthys alelia
